# 

**Published:** 1896-04-25

**Authors:** 


**Notice to Advertisers and Subscribers.**

All Advertisements, Orders for Copies of The Hospital and Business Communications should be addressed to THE MANAGER, (Not to The Editor), The Hospital, 428, Strand, London, W.C.

The Cost of Subscription, post free, is as follows:—Per week, 2½d.; per quarter, 2s. 8d.; per half-year, 5s. 3d.; per annum, 10s. 6d. Foreign:—Per Annum, 15s. 2d.; payable, in all cases, in advance.

Ready Shortly.

**"Burdett's Hospitals and Charities, 1896."**

Seventh year of publication. Over 1,000 pp. Scarlet cloth, gilt lettered. Price 5s. A most complete guide to British, Colonial, Indian, and American Hospitals, Dispensaries, Nursing and Convalescent Institutions, and Asylums. A few complete sets of the preceding six issues of the "Annual" may still be obtained on application to the Publishers, The Scientific Press (Limited), 428, Strand, London, W.C.

### Books Received.

Spottiswoode and Co.

"Medical Register, 1896."
"The Dentists' Register, 1896."

Hay, Nisbet, and Co.

"Hygienic Treatment for the Preservation of Health and Cure of Disease without Medicine," including an abridgment from a treatise by Dr. A. Wilford Hall, Ph.D., with introduction by the late Archibald Hunter.

Funk and Wagnalls Co. (New York).

"Youthful Eccentricity a Precursor of Crime." By Forbes Winslow.

Kegan Paul and Trench.

"Mediterranean Winter Resorts." By Eustace A. Reynolds Ball, F.R.G.S. Third edition.

**Periodicals and Pamphlets.**—Indian Medico-Chirurgical Review, New York Medical Journal, Literary Digest, Medical Pioneer, Medical Week, Medical Press, Vectis, Nursing Notes, Columbus Medical Journal, Humanity, Charlotte Medical Journal, Clinical Journal, London, Therapist, St. Louis Medical and Surgical Journal, Pediatrics, Canada Medical Record, Invention, Sisters, Photogram, Girl's Own Paper, Boy's Own Paper, Leisure Hour, Sunday at Home, Cornhill Magazine, Industries and Iron, Electrical Review, Cassell's Family Magazine, British and Colonial Druggist, Child's Guardian, Australian Medical Gazette, Planter, Sun, Medical Bulletin, Verulam Review, Practitioner, Guy's Hospital Gazette, Aluminium, Academy, Windsor Magazine, Humanitarian, Nursing Record, Clinical Sketches, Homœopathic Review, Leeds Hospital Magazine.

### The Student of Medicine.

### APPOINTMENTS.

C. Arrol, M.D., C.M.Glasg., appointed Medical Officer of Health for the Borough of Queensborough. C. H. W. Bennett, L.R.C.P.Lond., M.R.C.S.Eng., appointed Medical Officer of Health for the Congleton Rural District. J. W. Bird, M.R.C.S.Eng., L.R.C.P.Lond., appointed Junior Assistant House-Surgeon to Hull Royal Infirmary. S. W. Coombs, F.R.C.S.Edin., L.R.C.P.Edin., appointed Medical Officer for the Claines District of the Droitwich Union. T. V. Cunliffe, M.R.C.S.Eng., L.R.C.P.Lond., appointed House-Surgeon to the Manchester Southern Hospital for Women and Children, and to the Maternity Hospital in connection with it. S. B. Be Butts, M.D.Brux., M.R.C.S., etc., appointed Anæsthetist to the Grosvenor Hospital for Women and Children, Westminster. H. M. Eyres, M.B., C.M., appointed Resident Physician to the Edinburgh Royal Infirmary. C. Gibbs, F.R.C.S., appointed Assistant Surgeon to the Charing Cross Hospital. W. T. Hannah, M.B., C.M.Glasg., D.P.H.Camb., appointed Medical Officer and Public Vaccinator for the Buxton District of the Chapel-en-le-Frith Union. J. Healey, M.B., Ch.B.Vict., appointed Medical Officer of Health for Mossley, Lancashire. M. E. Kenny, B.A.Dubl., M.B., B.Ch., appointed Medical Officer for the Carrick Workhouse. W. S. Limerick, L.R.C.P., L.R.C.S.Edin., appointed Medical Officer of Health to the Waterloo-with-Seaforth Urban District Council. E. G. G. Little, B.A., M.D., M.R.C.P., appointed Assistant Curator of the Museum at St. George's Hospital. W. Macdonald, L.R.C.P., L.R.C.S.Edin., appointed Junior House-Surgeon to the Royal Albert Edward Infirmary, Wigan. C. Macpherson, M.D., appointed Deputy Commissioner in Lunacy for Scotland. H. S. Maw, L.S.A.Lond., appointed House-Surgeon to the Teignmouth Hospital. W. C. Milroy, M.A., M.D.Edin., appointed Senior House-Surgeon to the Royal Albert Edward Infirmary, Wigan. E. C. Moore, M.B., M.S.Edin., appointed Junior House-Surgeon to the Birmingham and Midland Eye Hospital. H. C. Pauli, appointed one of the Medical Officers of the Bute Hospital, Luton, Bedfordshire. F. S. Penny, M.R.C.S.Eng., L.R.C.P.Lond., of King's College Hospital, London, appointed House-Surgeon to the Infirmary, Sunderland. E. S. Pollock, B.A., M.B.Dub., B.Ch., appointed Medical Officer for the No. 6 District of the South Molton Union. G. A. Reid, M.B., C.M.Aberd., appointed Junior Medical Assistant to the Cumberland and Westmorland Asylum, Carlisle. L. W. Roberts, M.B.Melbourne, &c., appointed House Surgeon to the Victoria Hospital, Folkestone. J. I. Sankey, L.R.C.P.Lond., M.R.C.S.Eng., appointed Medical Officer for the Brenchley District of the Tonbridge Union. G. H. Savage, M.D.Lond., appointed Physician for Mental Diseases to Guy's Hospital. H. Scurfield, M.D.Edin., D.P.H.Camb., appointed Medical Officer of Health for Sunderland. L. M. Snow, M.R.C.S.Eng., L.S.A., appointed Medical Officer for the Second District of the Horsham Union. G. Vawdry, L.R.C.P.Edin., M.R.C.S.Eng., appointed Medical Officer of Health to the Farnborough Town Council. D. Walker, M.B., C.M.Aberd., appointed Junior Assistant Medical Officer to the Cornwall County Asylum.

### VACANCIES.

The following vacancies are announced this week, the amount of salary in each case being given after the name of the institution :—

### Resident Medical Officers.

Donegal District Lunatic Asylum, Letterkenny.—Assistant Medical Officer; qualified in medicine, surgery, and midwifery; unmarried, and not more than 30 years old. Salary £100 per annum, with furnished apartments, rations, washing fuel, light, and attendance, valued at £100 per annum. Applications to Dr. Moore, Resident Medical Superintendent, by May 9th.

General Hospital, Birmingham.—Assistant House Surgeon. Appointment for six months. No salary, but residence, board, and washing provided. Applications to Howard J. Collins, House Governor, by April 25th.

Hereford County and City Asylum.—Medical Superintendent. Salary £400 per annum, with furnished house, coals, gas, vegetables, and washing. Applications to the Chairman, Asylum Committee, Shirehall, Hereford, by April 28th.

Hospital for Sick Children, Great Ormond Street, Bloomsbury.—Surgical Registrar. Appointment for one year. Honorarium £40. Also House Physician; unmarried. Appointment for six months. Salary £20, with board and residence in the hospital. Applications to the Secretary by April 28th.

Huddersfield Infirmary.—Junior House Surgeon. Salary £40 per annum, with board, lodging, and washing. Appointment for one year. Applications to Mr. J. Bate, Secretary, Infirmary, Huddersfield, by April 27th.

Leicester Infirmary.—Assistant House Surgeon. Appointment for six months, subject to re-election. Honorarium of £21 for the six months, with board, residence, and washing. Applications to the Secretary, 24, Friar Lane, Leicester, by April 27th. Also Honorary Ophthalmic Surgeon. Applications to the Secretary by May 4th.

Macclesfield General Infirmary. — Junior House Surgeon; doubly qualified. Salary £70 per annum, with board and residence. Applications to the Chairman of the House Committee by April 30th.

North-West London Hospital, Kentish Town Road.—Resident Medical Officer and Assistant Resident Medical Officer. Appointment for six months. Salary at the rate of £50 per annum attached to the senior post. Applications to Alfred Craske, Secretary, by May 2nd.

South Devon and East Cornwall Hospital, Plymouth.—Assistant House Surgeon. Appointment for six months, subject to re-election. Board and residence provided, and an honorarium of £10. Applications to Hemsley H. Shanks, R.N., Honorary Secretary, by April 30th.

### Non-Resident Medical Officers.

Dental Hospital of London and London School of Dental Surgery, Leicester Square.—Lecturer on Mechanical Dentistry. Applications to Morton Smale, Dean, by May 11th.

Hastings, St. Leonard's, and East Sussex Hospital. — Honorary Ophthalmic Surgeon. Applications to the Secretary at the Hospital, White Rock, Hastings, by May 2nd.

Llantrissant and Llantwit Vardre Rural District Council.—Medical Officer. Salary £30 per annum. Applications to E. C. Spickett, Clerk to the Council, by April 27th.

Owens College, Manchester.—Junior Demonstrator in Anatomy and Junior Demonstrator of Physiology. Applications to the Registrar by April 27th.

Westminster Hospital, Broad Sanctuary, S.W.—Surgical Registrar; must be F. or M.R.C.S.Eng. Appointment for twelve months. Salary £40 per annum. Applications to Sidney M. Quennell, Secretary, by April 25th.

